# A Case of Dysentery

**Published:** 1888-02

**Authors:** J. K. Mendenhall

**Affiliations:** Saratoga, New York


### A CASE OF DYSENTERY.

REV. J. K. MENDENHALL, SARATOGA, NEW YORK.

Aug. 23d.—Was called to see S. H., a servant girl, age, eighteen; pulse, 100; profuse perspiration, severe pain in head, back, arms, legs, and abdomen, the latter extremely painful under slight pressure; stool very frequent and painful. Of this I could get no account save that it was excessively fetid and contained much blood. Constantly recurring chills.

HISTORY.—She had been working very hard all summer, and for two months had eaten large quantities of candy. Two and a half days previous to the attack she had eaten eighteen crab apples. On the morning of the 21st she had fainted on rising from bed. Her employer gave her at that time a pint of Citrate of Magnesia; this was soon followed by copious discharges from the bowels and excessive pain in them; fever also put in its appearance. Alarmed by this a Dover's powder was given, but the patient grew worse. During the night of the 22d fifteen drops of Aconite were given in about ten hours. It was at this juncture that I saw the patient; gave Nux vom.$^{1m}$ (B. &. T.), one dose, followed by Sac. Lac. Saw the patient again in the evening, condition much the same.

24th, 8.30 A.M.—Pulse, 98; no thirst, little perspiration; tongue yellowish white with *deep teeth marks;* abdomen *so tender she shrunk from the slightest touch;* stool frequent, exceedingly painful, and only *blood and milky water;* felt as though *she would never get done;* strength gone; abdominal pain constant. Merc. viv. 60 x trit. (B. & T.), 3 powders at intervals of two hours, followed by Sac. Lac.

8 P. M.—Slight amelioration of symptoms.

25th, 10 A. M.—Patient had slept some, stools less frequent, all symptoms better. Sac. Lac.

8 P. M.—Improvement continued.

26th, 9 A. M.—But two discharges from the bowels during the night, and these contained fecal matter; pains had largely disappeared, and patient could tolerate deep pressure on abdomen. Sac. Lac.

8 P. M.—Two discharges during the day of fecal matter alone; patient hungry, sat up while bed was made; no ill effects followed.

27th, 9 A. M.—A fair night, two movements of bowels, a little blood with fecal matter. Sac. Lac.

8 P. M.—Had a comfortable day, sat up an hour, felt better for it; *no pain* anywhere, very hungry.

28th, 7 P. M.—A comfortable day, no pain, discharges normal, good appetite, strength returning. Sac. Lac.

30th.—Patient sitting up all the time; good spirits, good appetite, strength returning, alvine discharges, normal in character and frequency, no trouble of any kind; patient anxious to resume work; cure complete.

